# Establishment and validation of an immune infiltration predictive model for ovarian cancer

**DOI:** 10.1186/s12920-023-01657-x

**Published:** 2023-09-28

**Authors:** Zhenxia Song, Jingwen Zhang, Yue Sun, Zhongmin Jiang, Xiaoning Liu

**Affiliations:** 1 Department of Obstetrics, Qingdao women and childeren’s hospital, #6 Tongfu Road, Shibei District, Qingdao, Shandong 266000 P. R. China; 2 Department of Pathology, Tian Jin Fifth’s Central Hospital, #41 Zhejiang Road, Binhai District, Tianjin, 300450 P. R. China

**Keywords:** TP53 mutation, Ovarian cancer, IPM, Immune infiltration

## Abstract

**Background:**

The most prevalent mutation in ovarian cancer is the TP53 mutation, which impacts the development and prognosis of the disease. We looked at how the TP53 mutation associates the immunophenotype of ovarian cancer and the prognosis of the disease.

**Methods:**

We investigated the state of TP53 mutations and expression profiles in culturally diverse groups and datasets and developed an immune infiltration predictive model relying on immune-associated genes differently expressed between TP53 WT and TP53 MUT ovarian cancer cases. We aimed to construct an immune infiltration predictive model (IPM) to enhance the prognosis of ovarian cancer and investigate the impact of the IPM on the immunological microenvironment.

**Results:**

TP53 mutagenesis affected the expression of seventy-seven immune response-associated genes. An IPM was implemented and evaluated on ovarian cancer patients to distinguish individuals with low- and high-IPM subgroups of poor survival. For diagnostic and therapeutic use, a nomogram is thus created. According to pathway enrichment analysis, the pathways of the human immune response and immune function abnormalities were the most associated functions and pathways with the IPM genes. Furthermore, patients in the high-risk group showed low proportions of macrophages M1, activated NK cells, CD8^+^ T cells, and higher CTLA-4, PD-1, PD-L1, and TIM-3 than patients in the low-risk group.

**Conclusions:**

The IPM model may identify high-risk patients and integrate other clinical parameters to predict their overall survival, suggesting it is a potential methodology for optimizing ovarian cancer prognosis.

**Supplementary Information:**

The online version contains supplementary material available at 10.1186/s12920-023-01657-x.

## Introduction

Ovarian serous cystadenocarcinoma (OV) is a fatal gynecological cancer in women, accounting for almost 14,000 new deaths in the United States in 2018 [1]. Biomarkers, particularly gene expression in tumor tissues, are consistently associated with cancer prognosis and survival [2, 3]. As a result, Researchers identified the subgroup of patients with poorer prognoses and increased death required for further therapeutic care. The availability of large-scale public cohorts with gene expression data, as well as a well-developed biology database, opens up the possibility of identifying a universal prognostic signature with a biological basis for ovarian cancer.

The immune system has been proven to have a role in cancer development and progression [4, 5]. Several studies have shown that ovarian cancer tumors are immunogenic [6, 7], and immunotherapy is being aggressively sought by targeting immunological checkpoints [8, 9]. Furthermore, earlier research has tentatively validated the immune system's predictive relevance in ovarian cancer [10]. As a result, the immune-based prognostic signature can be used in ovarian cancer.

Sentences like "TP53 is frequently mutated gene in human cancer" have featured in the introductions of hundreds of papers going back to 1990 [11, 12]. The TP53 mutation is often one of the five most prominent mutations in common human malignancies [13, 14]. The wild-type TP53 protein is vital in cell cycle regulation and apoptosis after cell injury [15]. On the other hand, cells with mutations may escape apoptosis and grow into malignant cells if TP53 is altered. According to a study that comprised 12 tumor types and 3281 patients, the mean alteration rate of TP53 was about 42% [13]. Because of TP53's high mutation rate, genetic change is a particularly appealing prospective therapeutic target. This gene is critical for genomic stability, and its functional loss may result in centrosome amplification, aneuploid cell proliferation, and chromosomal instability (CIN) [16]. TP53 mutations are more prone to increase CIN and genomic instability when paired with functional deficiencies in the tumor suppressive gene pRb or spindle checkpoint problems [17]. Considerable evidence suggests that TP53 mutation simultaneously abandons their tumor-suppressive capabilities while gaining new abilities to promote tumorigenesis [18].

This research aimed to determine the role of TP53 status in modifying the immunological signature in OV. A systematic review of the mutational landscape, expression profiles, and the relative abundance of tumor-infiltrating lymphocytes (TILs) from the multiple cohorts were performed in the present work to evaluate the functional deviations and immunotherapeutic landscape of OV. Immune-based biomarkers associated with the status of TP53 alterations were explored to develop and verify an immune infiltration predictive model (IPM). Furthermore, a nomogram incorporating either immunological markers or clinical aspects was created and tested to maximize their mutual efficacy. Our findings show that the IPM is an independent prognostic factor for OV patients and a good predictor for an immunotherapeutic response. The final integrated nomogram is very useful for doctors in quantitatively predicting the overall survival of OV patients.

## Materials and methods

### Data collection

The somatic mutation data, gene expression profile and clinical information of the ovarian cancer TCGA cohort were downloaded using the TCGAbiolinks package [19]. Testing cohorts were acquired from the Gene Expression Omnibus (GEO) under GSE13876, GSE30161, and GSE51088. Information involving the patients subjected to the immunotherapy was obtained from the published studies [20, 21]. The datasets used in this study were summarized in Table S1.

### Gene set enrichment analysis (GSEA)

To evaluate the immunological effect of TP53 mutation in ovarian cancer, the TCGA-OV cohort was divided into the TP53-WT (*n* = 248) and TP53-MUT (*n* = 26) subtypes depending on the TP53 mutation status. We performed GSEA between the two subtypes using the immunologic signature gene set (c7.all.v7.5.1.symbols.gmt) with permutations of 10^4^ [22]. *P*-value < 0.05 was defined as significant.

### Establishment and validation of the IPM

We developed the IPM based on a series of model algorithms from the immune-related genes better to evaluate ovarian cancer's overall survival and immunotherapeutic response. First, the significant differentially expressed genes (DEGs) were generated using the DESeq2 package between the TP53-WT and TP53-MUT subtypes in the TCGA-OV cohort. Then, the expression profiles of DEGs that overlapped with genes in the immunologic signature gene set were extracted as the immune-related genes for the following analysis. Second, the least absolute shrinkage and selection operator (LASSO) analysis was performed with the five-fold cross-validation to screen the hub genes. Third, the genes identified upwards of 900 occasions in 1000 repeats were retained. We constructed the IPM model using the regression coefficients originating from the multivariate Cox regression analysis. The suitable cutoff value was calculated using the “surv_cutpoint” function of survminer R package based on the risk score, overall survival status and times. Patients were divided into high- and low-risk groups using the same cutoff value obtained from the TCGA training cohort. Finally, the IPM was validated by three independent testing cohorts to ensure stringency. The Kaplan-Meier analysis and the log-rank test were used to assess the predictive ability; the time-dependent receiver operating characteristic (ROC) curve was applied to show the diagnostic ability under the different times.

### Estimation of immune cell infiltration

Immune cell fractions in the tumor microenvironment were adopted from the published study [23]. Four immunosuppressive cells were calculated using the TIDE [24] and xCell [25]. The Spearman correlation was estimated between the risk score and immunosuppressive cell infiltration. A *P*-value less than 0.05 is significant.

### Functional enrichment

We explored the functional deviations between the two IPM subgroups using the clusterProfiler package [26]. Benjamini-Hochberg (BH) adjusted *P*-value less than 0.05 was defined as significant. Metascape was used to construct the interactive network to present the functional characteristic [27].

### Construction and assessment of the nomogram model

Rms package ran a Univariable Cox analysis on diagnostics variables in the training dataset. Then, a multivariate Cox analysis was conducted using all significant (*P*-value < 0.05) variables, including age, race, TP53 status, and the IPM subgroup. Forestplot was used to depict the hazard rate and *P*-value of variables from the Cox analysis. A nomogram was drawn using rms package to help doctors assess the 1-year, 3-year, and 5-year OS probability of OV patients. To assess the outcomes' performance, discrimination and calibration analyses were used. The capacity to discriminate was assessed using a pec package and a time-dependent concordance index (C-index) calculated using the bootstrap method with 1000 resampling. The calibration was evaluated using rms package, which included mapping the nomogram predicted probability of 1-year, 3-year, and 5-year overall survival (OS) against the observed data rates.

### Chemotherapeutic response prediction

Based on the most extensive publicly released pharmacogenomics database (the Genomics of Drug Sensitivity in Cancer (GDSC), https://www.cancerrxgene.org/), we estimated the chemotherapeutic response for each sample. The assessment approach is carried out using the R package "pRRophetic," in which the half-maximal inhibitory concentration (IC50) of the samples was estimated by ridge regression, and the accuracy rate was evaluated utilizing 10-fold cross-validation based on the GDSC training set [28].

### Human sample collection

Human specimens containing 20 OV patients utilized in this investigation were acquired from patients undergoing surgical operations at Tian Jin Fifth’s Central Hospital and frozen at -80 °C for qRT-PCR. All materials were examined using HE staining in accordance with standard protocol, and two different pathologists made the diagnoses.

### qRT-PCR analysis

qRT-PCR was performed to validate the gene expression levels of our three target genes (TFPI, TNC, and ELK3). The high- and low-group were stratified using the median risk score calculated from the three target genes. Total RNA was extracted from the collected tissue samples using a commercially Trizol Reagent as described everywhere. cDNA synthesis was carried out using the PrimeScript RT Reagent Kit (Invitrogen, USA). qRT-PCR was performed by One-Step qPCR Kit (Invitrogen) and CFX ConnectTM Real-Time System (BIO-RAD) following manufacturer’s instructions. The relative gene expression levels were calculated using the ΔCt method with normalization to a reference gene GAPDH.

### Immunohistochemistry

Fresh target tissues were collected and fix it in 10% neutral buffered formalin for an appropriate period. Transfer the tissue into a labeled cassette and dehydrate it by placing it in a series of increasing concentrations of alcohol for gradual dehydration. Embed the tissue in molten paraffin wax and allow it to solidify. Then, the paraffin-embedded tissue block were cut to 4 μm section and subjected to deparaffinization and rehydration with xylene and graded alcohols. 3% H_2_O_2_ was utilized to eliminate endogenous peroxidase after antigen retrieval with five mM citrate buffer. The slides were blocked for 30 minutes at room temperature with goat serum before being treated with primary antibodies overnight at 4 ℃. The slices were rinsed three times in PBS before being treated for two hours at room temperature with a biotinylated secondary antibody. As a chromogen substrate, diaminobenzidine was utilized. Hematoxylin was used to counterstain the sections at the end. Immunohistochemistry was performed using antibodies against IL-17 (ab79056, Abcam), CD163 (ab79056, Abcam), CD64 (ab140779, Abcam), CD1A (17325-1-AP, Proteintech), CD57 (19401-1-AP, Proteintech), CD8 (66868-1-Ig, Proteintech) and CD3 (3F3A1, Proteintech).

### Statistical analysis

Fisher's exact test or Pearson's Chi-square test compared independent variables. For survival data analysis and preparation of Kaplan-Meier plots, the R packages survival and survminer were used. *P*-value < 0.05 was applied to determine statistical significance, and all statistical analysis was performed using R version 4.0.4 (https://www.r-project.org/).

## Results

### The association of TP53 mutations with immunophenotype in OV

**Fig. 1:**
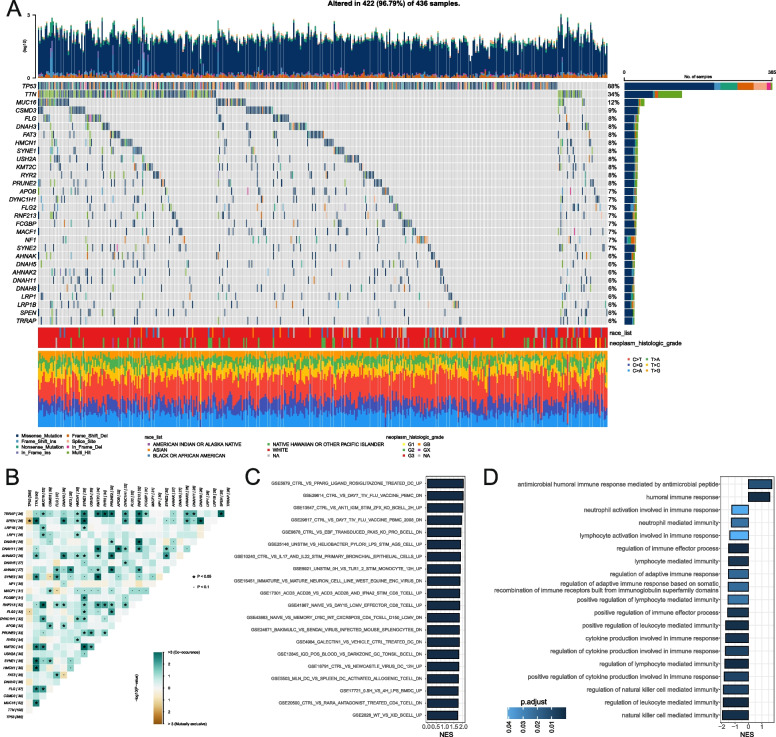
Somatic mutation spectrum and GSEA enrichment based on TP53 status in ovarian cancer. **A** Mutational landscape of top 30 genes in ovarian cancer. **B** Co-occurrence and mutual exclusivity analysis of the top 30 mutated genes. **C** Top 20 immune-related functions of TP53 mutational patients in ovarian cancer. **D** Immune-related functions of TP53 mutational patients analyzed by GO enrichment

The most prevalent mutation found in OV is TP53 (370/420, 88%; Fig. 1A). Co-occurrence and mutual exclusivity analysis indicated TP53 mutation co-occurred with SPEN and was mutually exclusive with other genes (*P*<0.05; Fig. 1B). TP53 mutations are related to the survival of OV patients, according to pioneering research [29]. Although TP53 mutations have a well-documented pathogenetic role in the outcome of patients with OV, their particular effects on immunological profiles in OV have not been thoroughly studied. As a result, we used expression profiles and medical data from OV patients in the TCGA for the first time to look for immune-related biochemical functions associated with TP53 status. OV samples with TP53 mutations (*n* = 248) and without TP53 mutations (*n* = 26) were subjected to GSEA analysis. TP53 MUT OVs were highly enriched in 105 immune-related biological processes (Fig. 1C; Table S2). To provide a more comprehensive understanding of the immune-related functions of TP53 mutational patients in OV, we performed Gene Ontology (GO) enrichment analysis (Fig. 1D), which showed that 17 out of 19 immune-related functions were inhibited in the TP53 mutational patients, indicating an immune-suppressed status. This finding highlights the potential importance of TP53 mutation status as a predictor of immune-related outcomes in OV.

### Definition of the IPM and valuation its predictive power in training cohort

**Fig. 2:**
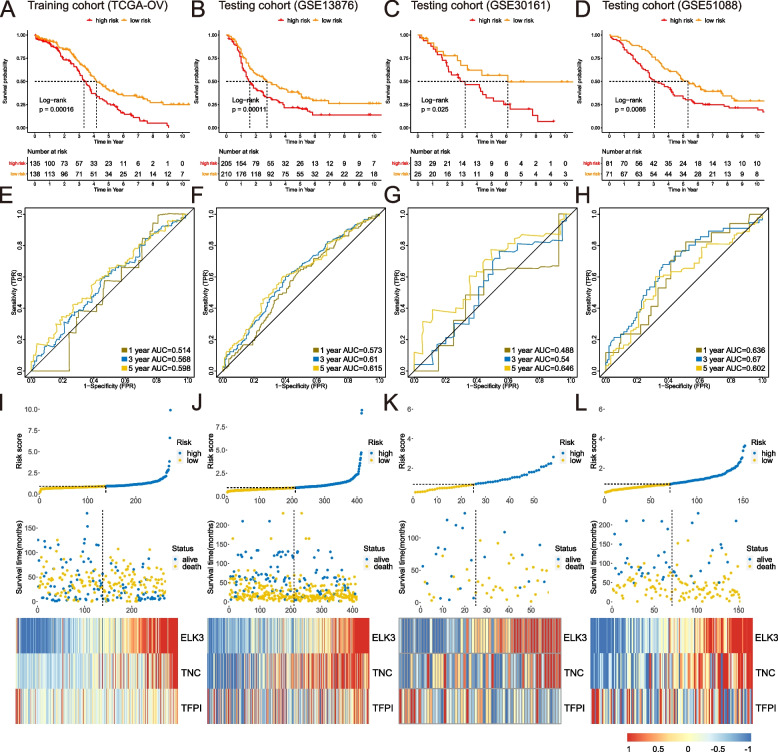
Establishment and validation of the IPM. Kaplan-Meier analysis for training cohort (**A**), and testing cohort GSE13876 (**B**), GSE30161 (**C**), and GSE51088 (**D**). Time-dependent ROC curve analysis for training cohort (**E**), and testing cohort GSE13876 (**F**), GSE30161 (**G**), and GSE51088 (**H**). Risk score assessment for training cohort (**I**) and testing cohort GSE13876 (**J**), GSE30161 (**K**), and GSE51088 (**L**)

In the TCGA training cohort, univariate Cox analysis investigated the connection between genes and OS (Table S3-4). The LASSO algorithm chose three DEGs (TFPI, TNC, and ELK3) that emerged more than 900 times out of 1000 repeats as necessary. Risk score depending on associated gene abundance adjusted by the Cox regression coefficients was produced using multivariate Cox analysis: IPM risk score = (ELK3 × 0.378) – (TFPI × 0.168) + (TNC × 0.196). Patients were divided into high- and low-risk groups using a feasible cutoff value (0.93). Figure 2A shows that high-risk individuals had a worse OS than low-risk patients (*P* = 0.00016). The AUCs of the IPM at 1-, 3-, and 5-years were 0.514, 0.568, and 0.598, respectively (Fig. 2E). Moreover, the expression levels of the three hallmark immunity genes were very compatible with the appropriate IPM risk score, with gene enrichment being more remarkable in the IPM high-risk subgroup in contrast to the IPM low-risk subgroup (Fig. 2I). To investigate the distribution of OV patients' status, we performed a chi-square test and found no significant difference between the high- and low-risk groups (*p* = 0.12, Figure S1A). Although not immediately evident, we observed a significant correlation between the risk score and survival times, providing valuable insights into the relationship between the risk model and patient outcomes (*p* = 0.019, Figure S1B).

### Verification of the IPM in three independent testing cohorts

Three independent validation testing cohorts from GEO datasets were used with the same equation to assess the IPM's resilience. Patients in the testing cohort were likewise divided into high- and low-risk subgroups using the same cutoff criterion (0.93). In testing cohorts, patients designated as low-risk had longer survival times than ones substantially from the high-risk subgroup (*P* < 0.05; Fig. 2B-D), confirming the findings of the TCGA training cohort. The AUCs have shown the validation cohort's predictive ability, demonstrating that the IPM is reliable across various datasets (Fig. 2F-H). Furthermore, heatmaps depict the correlation between the IPM and the expressed abundances of the three immunological hallmark genes in the three testing cohorts (Fig. 2J-L).

### IPM reflects the immunosuppression status in OV

**Fig. 3:**
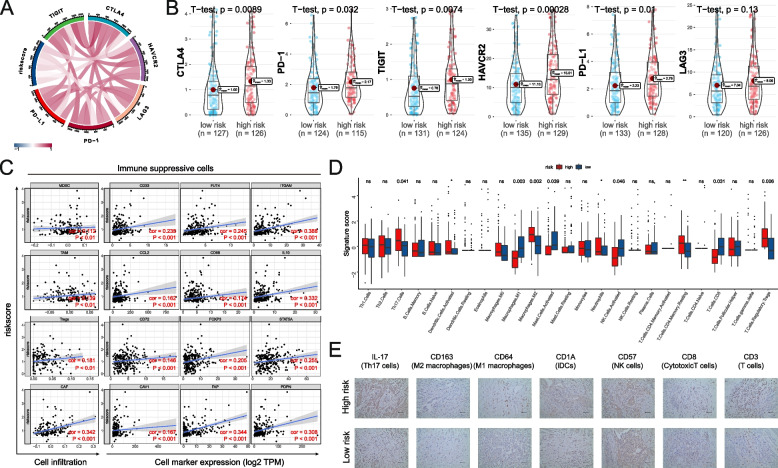
IPM correlates with immune suppression and CD8^+^ T cell exhaustion in ovarian cancer. **A** Spearman correlation between the risk score and six immunosuppressive genes. **B** Difference of six immunosuppressive genes between the high- and low- IPM groups. **C** Spearman correlation between the risk score and immunosuppressive cells and their representative markers. **D** The difference between the cancer immunity cycle signatures between the high- and low- IPM groups. **E** Representative IHC images of infiltrated immune cells in ovarian cancer

The immunosuppressive genes and cells in the TME are primarily responsible for the reduced percentage and dysfunction of CD8^+^ T cells. The association between IPM risk score and immunological checkpoints and immunosuppressive cells implicated in T cell depletion was then investigated. In OV, we discovered that the IPM risk score was linked to six immunosuppressive markers (PD-L1, PD-1, CTLA4, LAG3, HAVCR2, and TIGHT) (Fig. 3A; Table S5). These immunological checkpoints are essential in T cell activation and cause T cell function to regress [30]. Consistent with this notion, patents from the IPM high-risk group presented the high expression of these immunosuppressive markers compared with the low-risk patients (Fig. 3B).

Myeloid-derived suppressor cells (MDSC), tumor-associated macrophages (TAM), cancer-associated fibroblasts (CAF), and regulatory T cells (Treg) are four immunosuppressive cells that can prevent immune cells, particularly CD8^+^ T cells, from infiltrating the TME and suppressing their functions within the tumor [31]. Four immunosuppressive cells and their corresponding markers were shown to be substantially associated with the IPM risk score (Fig. 3C). Furthermore, we calculated the variations in immune recruitment of 25 immune cell types between the two IPM subgroups. The number of immune cells in OV varies across and among groups (Fig. 3D). As a result, alterations in the quantities of TILs could be an innate trait that distinguishes individuals. Th17 cells, macrophages M2, activated Dendritic cells, Neutrophils, T cells CD4 memory resting, and T cells regulatory (Tregs) were found in significantly larger percentages in high-risk OV patients. In contrast, macrophages M1 activated NK cells and CD8^+^ T cells were found in markedly decreased ratios in low-risk OV patients. Finally, the representative markers of specific immune cells were validated using immunohistochemistry in OV tissues as previously reported [32] (Fig. 3E).

### Functional deviations between IPM subgroups

**Fig. 4:**
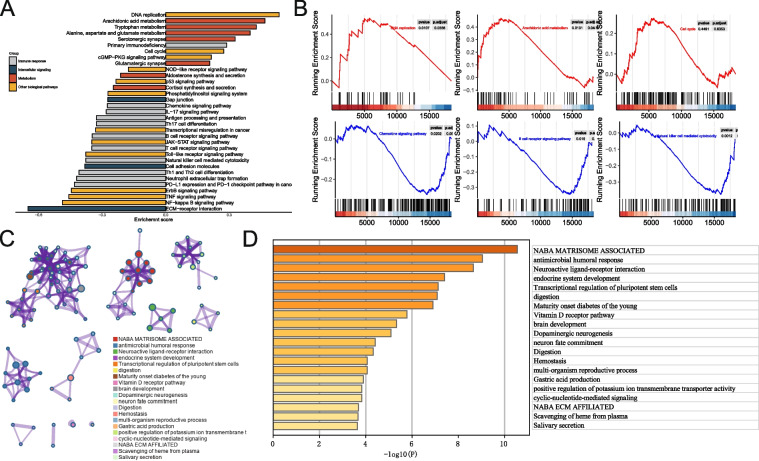
Functional deviations between the high- and low- IPM groups. **A** Differential activities of pathways between the high- and low- IPM groups. **B** Representative pathways between the high- and low- IPM groups. **C** Network of GO and KEGG enriched terms colored according to clusters. **D** Network of GO and KEGG enriched terms demonstrated according to *P*-values

According to the enrichment assessments, various cancer hallmark-related pathways, including immune response, intercellular communication, metabolism, and other physiological functions, were shown to differ significantly between the IPM high- and low- subgroups (Fig. 4A). The DNA replication, Arachidonic acid metabolism, Primary immunodeficiency, cGMP-PKG signaling pathway, and Cell cycle were activated substantially (Fig. 4A-B; FDR < 0.05). ECM-receptor interaction, Chemokine signaling pathway, B cell receptor signaling pathway, p53 signaling pathway, and Natural killer cell-mediated cytotoxicity were all down-regulated significantly (Fig. 4A-B; FDR < 0.05). Metascape was also used to investigate the physiological activities enrichment of IPM. The GO and KEGG terms network were colored according to the cluster and *P*-values (Fig. 4C-D; Table S6) [33]. IPM was involved in anticancer immune response, which may progress the immunosuppressive milieu of OV, a clear example of inflammation-related malignancy, according to the GSEA findings.

### Construction and evaluation of the nomogram according to the IPM

**Fig. 5:**
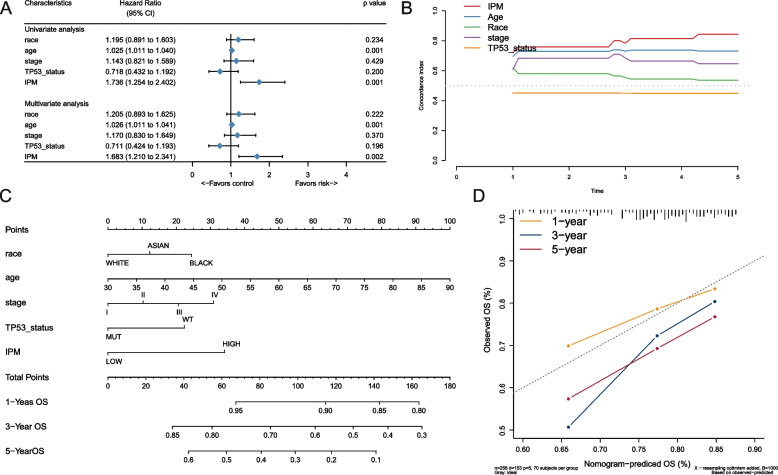
Comparation of IPM and conventional clinical characteristics. **A** Univariate and multivariate Cox regression analysis of IPM and some conventional clinical covariates in ovarian cancer patients. **B** Concordance index plot of IPM and some conventional clinical covariates. **C** Nomogram for overall survival at 1-, 3-, and 5-year in ovarian cancer patients. **D** Calibration plot at 1-, 3-, and 5-year for validation to predict overall survival probability

Univariate and multivariate Cox analyses were done progressively to determine whether the IPM was an independent prognostic factor, including the IPM risk score and other available clinical hazard variables such as age, race, stage, and TP53 mutations. The IPM was significant (*P* < 0.05) across either the univariate or multivariate Cox analyses, as shown in Fig. 5A, demonstrating that the IPM was practical to prognosticate the OS of OV patients severally. The multivariate cox analysis, the IPM presented the worst hazard rate (HR = 1.683, 95 percent CI = 1.210 – 2.341; Fig. 5A) within the considered covariates. To address how TP53 status correlates with IPM, we have calculated the IPM risk score for each patient in TCGA dataset, including both TP53 MUT and TP53 WT patients. We observed the significant correlation between risk score and TP53 status (Figure S3). In addition, Fig. 5B showed that the IPM had a greater time-dependent C-index (0.787) than all other clinical characteristics (0.415 – 0.765). In general, our findings showed that the IPM was an independent prognostic factor that outperformed other clinical characteristics in predicting the outcome of OV patients.

To increase the predictive accuracy of patients' OS using the IPM model, a nomogram was created by the TCGA cohort, including the IPM risk score and available clinical risk variables (Fig. 5C). Based on the multivariate Cox analysis, each covariate was assigned a score, and the total points nomogram score was obtained by adding the risk point values of all the covariates. By matching the total points to the 1-, 3-, and 5-years OS axes, the incidence of OS at 1-, 3-, and 5- years was estimated. When contrasted to other clinical criteria, the IPM presented the highest scores (coverage from 0 to 52). To assess the effectiveness of the nomogram, calibration plots (Fig. 5D) were created, which showed the estimated rates versus the actual rates at 1-, 3-, and 5-years intervals, indicating high stability. To assess the impact of the IPM on the nomogram, we generated a new nomogram without the IPM and compared it with the original version. Figure S2 shows the calibration plot of the nomogram without the IPM. We found that the inclusion of IPM in the nomogram improved its predictive accuracy and provided additional prognostic information, demonstrating that IPM is a critical component of the model. These data revealed that the nomogram is a better model than individual prognostic variables for predicting the survival time in OV.

### IPM predicts the immunotherapeutic response

**Fig. 6:**
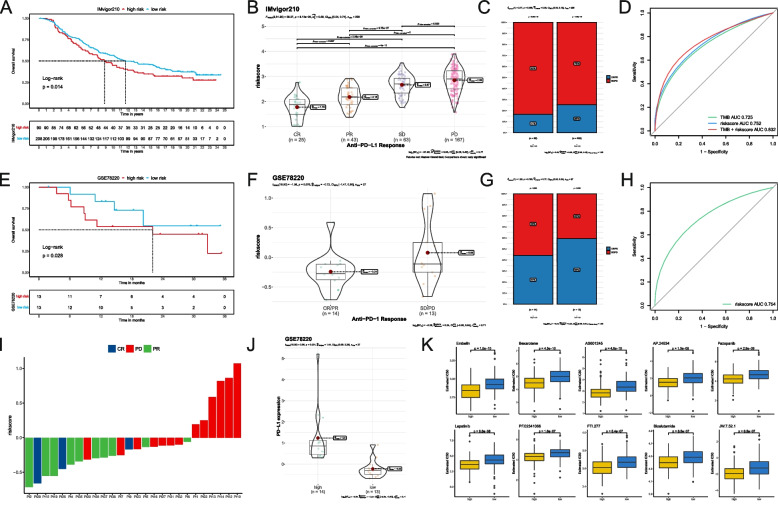
IPM predicts immunotherapeutic prognosis. **A** Kaplan-Meier analysis between the high-- and low- IPM group in the IMvigor210 cohort. **B** Risk score distribution for different anti-PD-L1 clinical responses in IMvigor210 cohort. **C** The relative proportion of clinical response to anti-PD-L1 immunotherapy in the high- and low- IPM group in IMvigor210 cohort (PD-progressive disease, SD-stable disease, PR-partial response, and CR-complete response). **D** ROC curves of TMB, risk score, and combination of TMB and risk score in IMvifor210 cohort. **E** Kaplan-Meier analysis between the high- and low- IPM group in GSE78220 cohort, (**F**) Risk score distribution for different anti-PD-1 clinical responses in GSE78220 cohort. **G** The relative proportion of clinical response to anti-PD-1 immunotherapy in the high- and low- IPM group in GSE78220 cohort. **H** ROC curves of the risk score in GSE78220 cohort. **I**)The risk score of each patient in GSE78220 cohort. **J** PD-L1 gene expression levels in the high- and low- IPM group. **K** The box plots of the estimated IC50 for the top 10 compounds in the high- and low- IPM group

Using the IMvifor210 and GSE78220 cohorts, we then investigated the predictive significance of the IPM for immune checkpoint inhibitor (ICI) treatment. In both the IMvifor210 and GSE78220 cohorts, patents with high IPM risk scores had a significantly worse prognosis than those with low IPM scores (Fig. 6A, E). ICI therapy may assist patients with low IPM low subgroup better (Fig. 6B-C, F-G). ROC was also used to measure tumor mutation burden (TMB), which is highly associated to immunotherapy efficacy. Nonetheless, we discovered that the IPM risk score alone had less predictive value than the TMB. However, combining TMB and IPM risk scores boosted predictive power compared to utilizing TMB or IPM alone (Fig. 6D, H). Figure 6I depicts the risk score for each sample. Since both scores combined improve the predictive value for IMvigor210 cohort, combining IPM with TMB into a single nomogram would be a valuable next step in validating our findings and exploring their clinical utility (Figure S4).

Furthermore, we compared the expression profiles of the two IPM subgroups to those of another cohort of 47 melanoma patients who responded to immunotherapies. Surprisingly, the expression of PD-L1 was higher in patients from the high IPM subgroup than those from the low IPM subgroup (Fig. 6J). Since chemotherapeutic therapy is a primary tool in tumor therapy, we predicted the response of commonly used drugs between the two IPM subgroups of patients using the GDSC dataset. Ten compounds exhibited an excellent response in IPM low subgroup (Fig. 6K).

## Discussion

Patients with high-grade ovarian serous carcinomas with wild-type TP53 seemed to have a worse prognosis and were more resistant to chemotherapy than those with mutant TP53 [29]. However, the prognostic significance of TP53 mutations and TP53 mutation types in ovarian cancer is debatable [34–36]. A meta-analysis reveals a broad range of variation in methodologies used to assess TP53 mutation status and categorize TP53 mutations into functionally significant categories [36]. Kang et al. found that TCGA cohort patients with TP53 mutations were more likely to develop distant metastases [37]. Consistent with Laxmi et al, there is no significant correlation was seen between TP53 mutations and clinical outcome [34, 38].

Distinct gene expression patterns in these TP53 wild-type tumors may illuminate the molecular mechanism behind treatment resistance [29]. Recent research has demonstrated that TP53 mutations can boost immunological checkpoint gene expression, trigger T effector cells, and increase interferon-γ in lung cancer [39]. Patients with co-occurrence mutations of TP53 and KRAS benefited more from blockers targeting PD-1 [39]. Anti-PD-1 antibodies have been shown to boost anti-tumor immunity in ovarian cancer [40]. However, the molecular mechanism by which TP53 mutations regulate the immunophenotype and prognosis of OV is unknown. As a result, the immunological implications of TP53 status in OV should be investigated.

Additionally, improved IPM was beneficial for identifying biomarkers, assessing the immunological status for OV sufferers, and classifying them to improve immunotherapy efficacy. Cancer immune-associated signatures have been created and discovered in multiple malignancies in recent years [41, 42]. While several analyses have tried to delineate immune-associated markers in OV [43–45], the true nature of the TME in OV outcome and prediction remains unknown. The functional enrichment revealed that the group with mutant TP53 had a considerably weaker immunological profile than wild-type TP53. Following that, a cox-proportional hazards analysis using the L1-penalized LASSO estimator identified three essential genes (TFPI, TNC, and ELK3) that were utilized to develop a unique IPM for OV patients.

TFPI, TNC, and ELK3 are less studied in OV, but have been well researched in other cancer. TFPI has been found to play a complex role in cancer, with both tumor-suppressive and tumor-promoting effects depending on the context [46]. TFPI can interact with various cellular signaling pathways implicated in cancer. It has been shown to interact with growth factor receptors, extracellular matrix proteins, and proteases involved in tumor invasion and metastasis. These interactions may influence tumor cell behavior and signaling cascades associated with cancer progression [47]. TNC gene is involved in cancer and has been found to play various roles in tumorigenesis and tumor progression [48, 49]. TNC is an extracellular matrix (ECM) protein that is upregulated in many types of cancer. It contributes to ECM remodeling by interacting with other ECM components, such as fibronectin and collagens. TNC promotes tumor cell migration, invasion, and metastasis by modulating the physical properties of the ECM [49]. Moreover, TNC interacts with cell surface receptors, including integrins, to promote tumor cell adhesion and migration. It can provide a permissive environment for cancer cells to move through the ECM and invade surrounding tissues. TNC has also been associated with epithelial-mesenchymal transition (EMT), a process linked to increased cancer cell motility and invasiveness [50]. The ELK3 gene, also known as NET (Nuclear Protein Related to SAM Pointed Domain-Containing ETS Transcription Factor), has been found to play roles in cancer development and progression [51–53]. It binds to specific DNA sequences and either activates or represses gene transcription. ELK3 can modulate genes related to cell proliferation, survival, angiogenesis, and metastasis, which are crucial processes in cancer development [52]. Alterations in ELK3 expression have been observed in various cancers, and its expression levels have been investigated as potential prognostic markers. Both increased and decreased ELK3 expression have been associated with poor prognosis in different cancer types, indicating its potential as a prognostic indicator [54].

We established via various methods that our IPM was more predictive of survival than typical clinical characteristics. Moreover, we conducted a complete review that considered both the IPM and traditional clinical parameters. The calibration curve demonstrated a strong level of concordance between the predicted and observed clinical features at 1-, 3-, and 5- years OS. Our primary advantage provides a unique viewpoint on tumors prognosis and offers a grading system for OV patients. As a result, the nomogram can be a valuable method for doctors. Elimination is a more sophisticated pattern of cancer immunosurveillance. The innate and adaptive immune systems work in concert to detect the presence of tumor growth and destroy it before it progresses [41]. IFNs may increase tumor development, stimulate dendritic cells, and enhance adaptive immune responses [55]. The adaptive immune system controls cancer cell expansion and shapes the tumor immunogenicity during equilibrium. The retention of occult tumor cells is facilitated by IL-12, IFN^-^, CD4^+^, and CD8^+^ T cells [56, 57].

The immunoediting process against cancer is divided into three stages: elimination, balance, and escape [56]. Variant cells that have progressed through the previous two stages and gained the capacity to evade immune detection enter the escape stage when they become visible and continue to proliferate. Thus, tumor escape occurs when an immunosuppressive state is established inside the tumor microenvironment [56, 58]. Tregs are a significant class of immunosuppressive cells that decrease host-protective anti-tumor responses. Tregs that have been activated may express PD-1, PD-L1, and CTLA-4 to suppress the activity of tumor-specific T cells [59]. We examined the immune infiltration of 25 immune cell types in low- and high-risk OV patients to better understand the immunological processes at work and assess the potential reach of the proposed IPM as cancer immunotherapy. The findings indicated that patients with high-risk OV had more Th17 cells, macrophages M2, activated Dendritic cells, Neutrophils, T cells CD4 memory resting, and Tregs.

Additionally, the high-risk OV subgroup's PD-1, PD-L1, LAG3, HAVCR2, and TIGIT gene expression levels were considerably more significant than low-risk OV patients. As a result, the IPM was compatible with immune infiltration's capacity to determine ICI treatment gene expression. We show that the high-risk individuals' worse prognosis may have been caused by a more favorable immunosuppressive milieu and greater expression of immune checkpoint genes. Overall, our study suggests that the high-risk group of patients with ovarian cancer have more immunosuppressive cells and higher expression of immunosuppressive molecules. However, the low-risk group responds better to ICI therapy. One possible explanation for this is that ICI therapy targets specific immune checkpoints, while the overall immune suppression in high-risk patients is driven by multiple factors, not all of which are targeted by ICI. Other factors that could influence the response to ICI therapy include tumor mutational burden, neoantigen load, and the presence of immunosuppressive stromal cells [60, 61]. Further research is needed to fully understand the underlying mechanisms. Nevertheless, our findings suggest that the inclusion of ICI genes in the IPM may provide a more accurate prognosis for high IPM scored OV patients, which could lead to significant advancements in prognostication.

## Conclusion

Given that the model reported here has relied on retrospective data, they should be additionally confirmed by potential investigations. Notably, the three critical immune-related genes employed to design the IPM should be identified in experimental trials to confirm their medical use. Moreover, this study adds to our understanding of OV's immunological milieu and immune-associated therapy. An IPM based on three immune-related genes were suggested for the first time. The suggested IPM was shown to be a significant predictive factor for OV patients and provided an overview of the immune response landscape in the OV microenvironment. Interestingly, the creation and assessment of the IPM provided an immunologic viewpoint for elucidating the processes behind the clinical outcomes of OV and may serve as a model for other forms of cancer.

### Supplementary Information

**Additional file 1: Figure S1.** Correlatio between IPM and patient survival. (A) Status distribution in high- and low group. (B) Correlation between risk score and survival.

**Additional file 2: Figure S2.** Calibration plot at 1-, 3-, and 5-year of nomogram without IPM.

**Additional file 3: Figure S3.** Distuibution of risk score in TP53 status.

**Additional file 4: Figure S4.** Nomogram for overall survival at 1-, 3-, and 5-year in ovarian cancer patients in IMvigor210 cohort.

**Additional file 5:** **Table S1.** Clinical information of TCGA-OV patients. **Table S2.** Immune-related genesets enriched in TP53 MUT OVs. **Table S3.** Differentially expressed immune-related genes between TP53 WT and TP53 MUT OVs.**Table S4.** Univariate Cox regression analysis of differentially expressed immune-related genes. **Table S5.** Analysis of correlations between risk score and immune checkpoints. **Table S6.** Top 20 clusters with their representative enriched terms by Metascape.

## Data Availability

The datasets used and/or analysed during the current study available from the corresponding author on reasonable request.
