# Supplementary material for: Establishment and validation of an immune infiltration predictive model for ovarian cancer
Source: BMC Med Genomics. 2023 Sep 28;16:227. doi: 10.1186/s12920-023-01657-x (PMC10538244; doi:10.1186/s12920-023-01657-x)
Supplement: Supplementary file 1 — Additional file 1: Figure S1. Correlatio between IPM and patient survival. (A) Status distribution in high- and low group. (B) Correlation between risk score and survival. [file 12920_2023_1657_MOESM1_ESM.pdf]

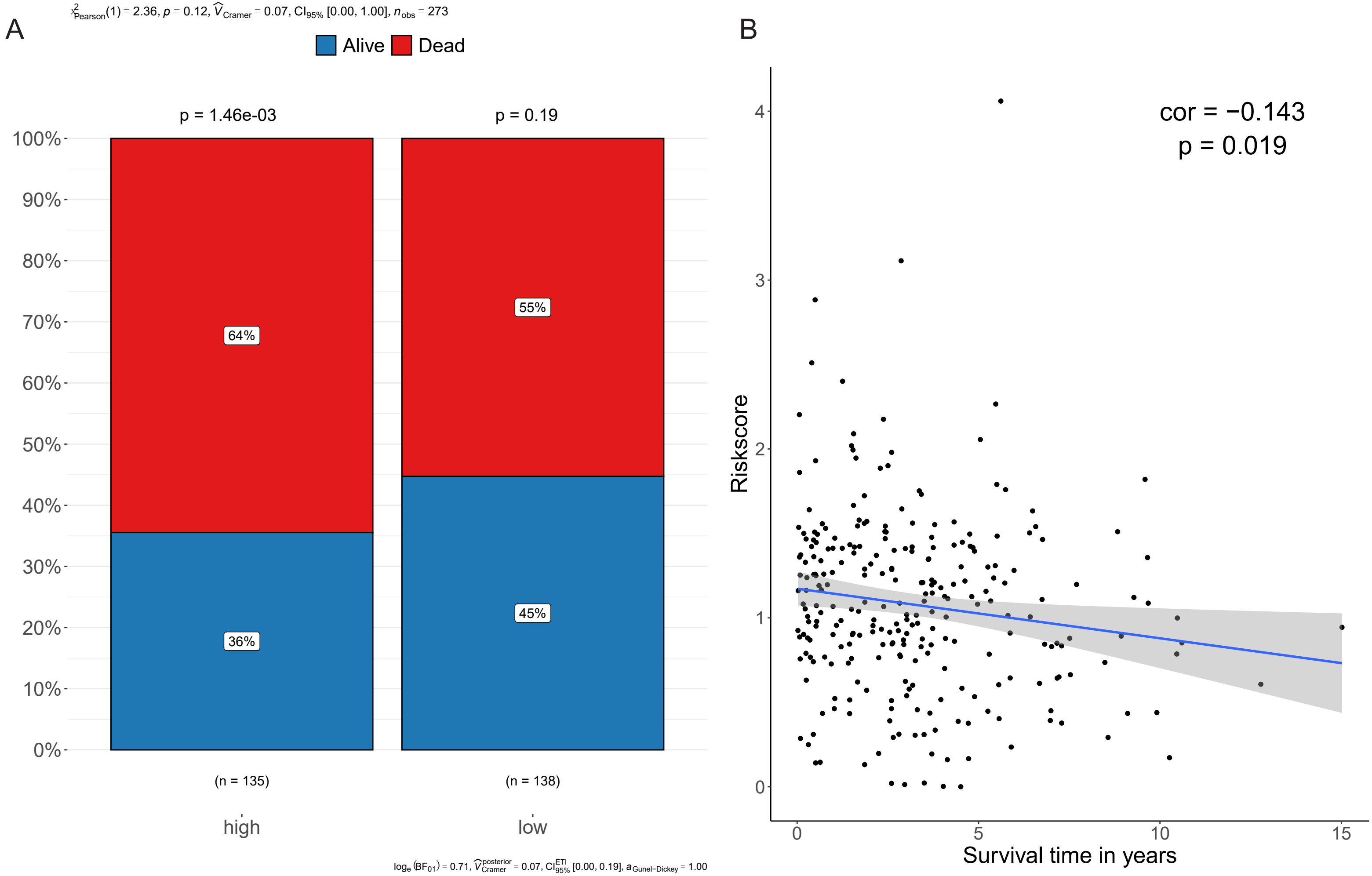

Figure S1. Correlatio between IPM and patient survival. (A) Status distribution in high- and low group. (B) Correlation between risk score and survival.
