# Supplementary figures and images for: Establishment and validation of an immune infiltration predictive model for ovarian cancer
Source: BMC Med Genomics. 2023 Sep 28;16:227. doi: 10.1186/s12920-023-01657-x (PMC10538244; doi:10.1186/s12920-023-01657-x)

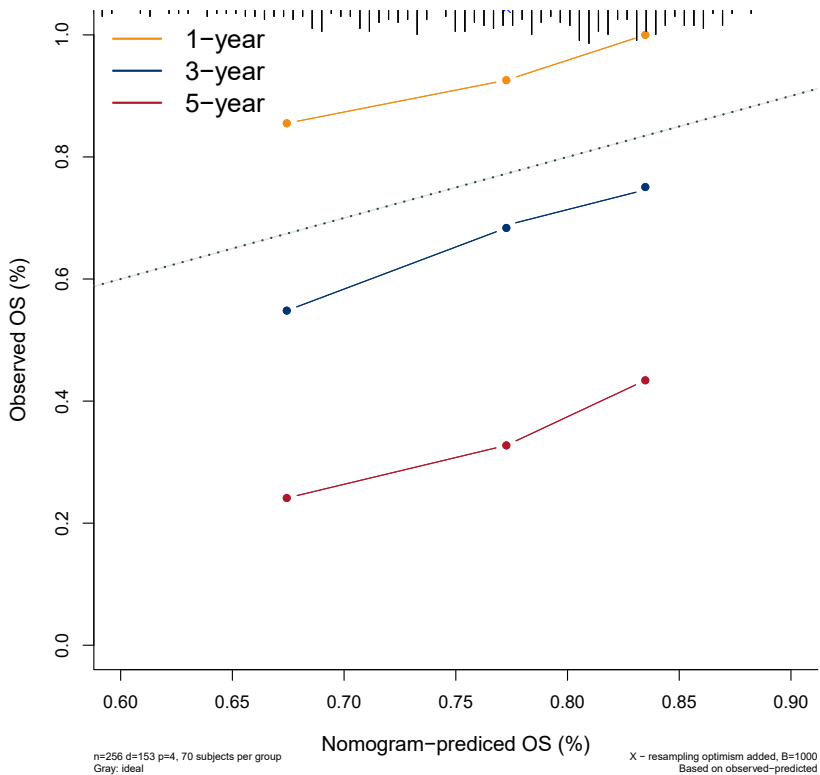

Figure S2. Calibration plot at 1-, 3-, and 5-year of nomogram without IPM.

Supplement: Supplementary file 2 — Additional file 2: Figure S2. Calibration plot at 1-, 3-, and 5-year of nomogram without IPM. [file 12920_2023_1657_MOESM2_ESM.pdf]

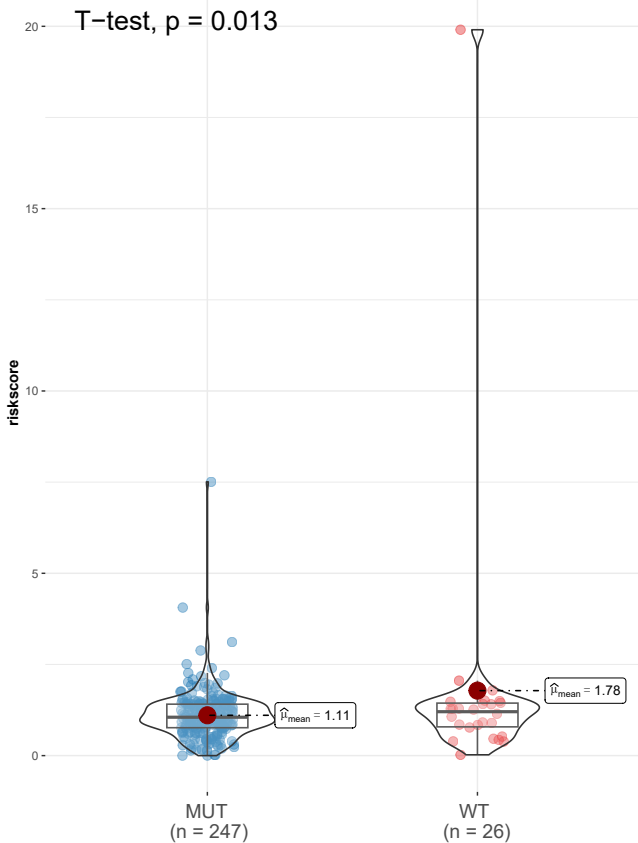

Figure S3. Distribution of risk score in TP53 status.

Supplement: Supplementary file 3 — Additional file 3: Figure S3. Distuibution of risk score in TP53 status. [file 12920_2023_1657_MOESM3_ESM.pdf]
