# Supplementary material for: Establishment and validation of an immune infiltration predictive model for ovarian cancer
Source: BMC Med Genomics. 2023 Sep 28;16:227. doi: 10.1186/s12920-023-01657-x (PMC10538244; doi:10.1186/s12920-023-01657-x)
Supplement: Supplementary file 4 — Additional file 4: Figure S4. Nomogram for overall survival at 1-, 3-, and 5-year in ovarian cancer patients in IMvigor210 cohort. [file 12920_2023_1657_MOESM4_ESM.pdf]

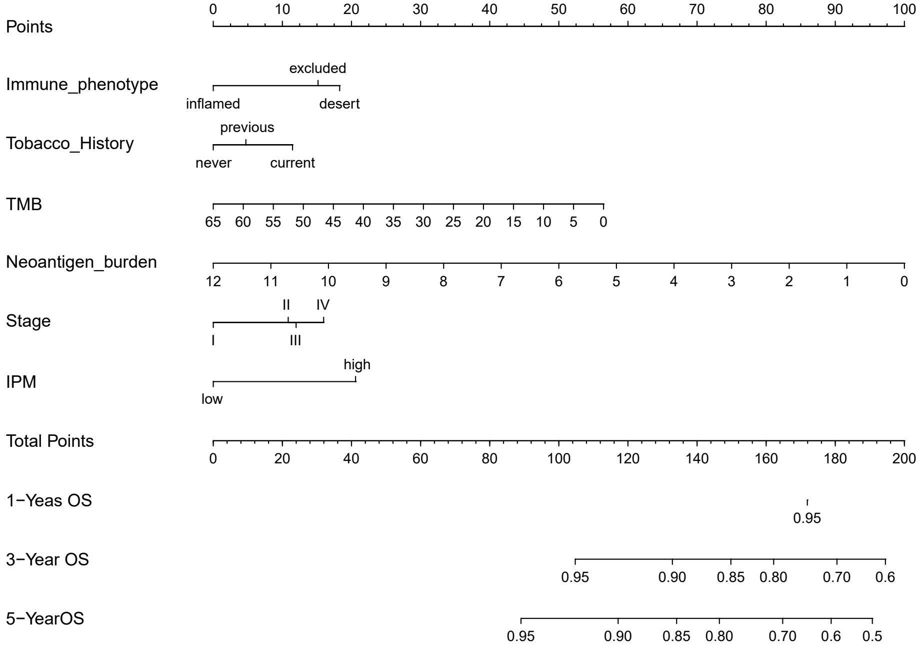


Figure S4. Nomogram for overall survival at 1-, 3-, and 5-year in ovarian cancer patients in IMvigor210 cohort.
